# Suppression of USP7 induces BCR-ABL degradation and chronic myelogenous leukemia cell apoptosis

**DOI:** 10.1038/s41419-021-03732-6

**Published:** 2021-05-07

**Authors:** Shuoyi Jiang, Xiaoge Wang, Yuanming He, Hongbiao Huang, Biyin Cao, Zubin Zhang, Jinbao Liu, Qi Wang, Zhenqian Huang, Xinliang Mao

**Affiliations:** 1 Department of Hematology, the First Affiliated Hospital of Guangzhou Medical University, Guangzhou, 510120 China; 2 Guangdong and Guangzhou Key Laboratory of Protein Modification and Degradation, School of Basic Medical Sciences, Guangzhou Medical University, Guangzhou, 511436 P. R. China; 3 Department of Pharmacology, Soochow University, Jiangsu, 215123 P. R. China; 4 Institute of Clinical Pharmacology, Science and Technology Innovation Center, Guangzhou University of Chinese Medicine, Guangzhou, 510405 China

**Keywords:** Deubiquitylating enzymes, Leukaemia

## Abstract

Chronic myelogenous leukemia (CML) is a clonal malignancy of hematopoietic stem cells featured with the fusion protein kinase BCR-ABL. To elicit the mechanism underlying BCR-ABL stability, we perform a screen against a panel of deubiquitinating enzymes (DUBs) and find that the ubiquitin-specific protease 7 (USP7) drastically stabilizes the BCR-ABL fusion protein. Further studies show that USP7 interacts with BCR-ABL and blocks its polyubiquitination and degradation. Moreover, USP7 knockdown triggers BCR-ABL degradation and suppresses its downstream signaling transduction. In line with this finding, genetic or chemical inhibition of USP7 leads to BCR-ABL protein degradation, suppresses BCR/ABL signaling, and induces CML cell apoptosis. Furthermore, we find the antimalarial artesunate (ART) significantly inhibits USP7/BCR-ABL interaction, thereby promoting BCR-ABL degradation and inducing CML cell death. This study thus identifies USP7 as a putative Dub of BCR-ABL and provides a rationale in targeting USP7/BCR-ABL for the treatment of CML.

## Introduction

Chronic myelogenous leukemia (CML) is a clonal myeloproliferative malignancy derived from the hematopoietic stem cells of bone marrow^1^. The genetic feature in CML is the formation of Philadelphia (Ph) chromosome by the translocation between Chromosomes 9 and 22, which results in the expression of the BCR-ABL fusion gene (the C terminus of the proto-oncogene kinase ABL juxtaposed to the N terminus of the BCR)^2,3^. In physiological status, the ABL kinase shuttles between the cytoplasm and the nucleus and displays both promotive and suppressive functions in cell proliferation and survival upon the context signals, however, the BCR-ABL fusion protein mainly retains in the cytoplasm and displays constitutive and abnormal tyrosine kinase activity^4^. As an oncogenic non-receptor kinase, BCR-ABL interplays with and aberrantly activates a panel of oncogenic cytoplasmic signaling molecules including Ras–mitogen-activated protein kinase (MAPK), the Janus-activated kinase (JAK)–STAT pathway, and the phosphoinositide 3-kinase (PI3K)/AKT pathway. BCR-ABL therefore promotes CML cell proliferation, survival and anti-apoptosis^4–6^. BCR-ABL is considered to be the fundamental event in CML pathogenesis and it is also the ideal therapeutic target for CML^7^. Imatinib (IM) has been widely used for the treatment of CML patients by selectively inhibiting BCR-ABL^8^.

The activity and stability of the BCR-ABL protein is modulated by several critical posttranslational modifications including ubiquitination, SUMOylation, phosphorylation, neddylation, and acetylation^9^. It has demonstrated that the BCR-ABL protein can be degraded through the ubiquitin-proteasome pathway (UPP)^10^. The protein ubiquitination is a process involved in the ubiquitin-activating enzyme, the ubiquitin-conjugating enzyme, and the ubiquitin ligase. It has been reported that the ubiquitin ligases CHIP^11^, c-CBL, and SH2-U-box^12^ induce the polyubiquitination of BCR-ABL and subsequent degradation. However, protein ubiquitination is a dynamic process and the conjugated ubiquitin molecules could be removed by a certain enzyme called deubiquitinase (Dub). In addition to USP25, a putative Dub recently identified for BCR-ABL deubiquitination^13^, some Dubs such as HAUSP (USP7) and USP9x are found to be associated with BCR-ABL^14^, and an USP9x inhibitor inactivates BCR-ACBL but does not modulate its stability^15^, whether these Dubs are also involved in BCR-ABL ubiquitination are not known.

In the present study, we found that USP7 as a Dub interacts with BCR-ABL and prevents it from K48-linked polyubiquitination and from proteasomal degradation. Consistently, USP7 activates the BCR-ABL signaling pathway and markedly increases CML cell viability. We also identified the antimalarial artesunate (ART) induces the degradation of BCR-ABL and CML cell death via inhibiting the interaction between USP7 and BCR-ABL.

## Materials and Methods

### Cell culture

Human embryonic kidney cells (HEK293T) were maintained in Dulbecco’s modified Eagle’s medium (DMEM). Leukemia cell line K562 was obtained from American Type Culture Collection (ATCC, Manassas, VA, USA). KBM5 cells^16,17^ were maintained in the lab. All cells were cultured in 10% fetal bovine serum (ExCell Bio, Inc., Shanghai, China) and appropriate antibiotics.

### Plasmids

A BCR-ABL plasmid was a generous gift from Dr. Yun Zhao, Soochow University, Suzhou, China. All Dub plasmids were obtained from Dr. Hui Zheng, Soochow University^18^. The pcDNA3.1-USP7 and lentiviral USP7 plasmids were prepared as reported previously^19^.

### Chemicals and antibodies

Antibodies against USP7, BCR-ABL, Lyn, p-Lyn (Tyr507), STAT5, p-STAT5 (Tyr94), PARP, CRKL, and p-CRKL (Tyr207) were purchased from Cell Signaling Technologies, Inc., (Danvers, MA, USA). The antibodies against USP25 and Caspase-3 were obtained from Proteintech (Wuhan, China). The monoclonal antibodies including anti-Flag, anti-HA, and anti-glyceraldehyde-3-phosphate dehydrogenase (GAPDH) were obtained from Medical and Biological Laboratories Co., Ltd (Nagoya, Japan). The anti-Ub antibody was purchased from Santa Cruz Biotechnology, Inc., (Santa Cruz, CA, USA). HRP-labeled goat anti-mouse and goat anti-rabbit IgG (H + L) antibodies were purchased from Beyotime Institute of Biotechnology (Nantong, China). ART and cycloheximide (CHX) were purchased from TargetMol (Boston, MA, USA), Sigma-Aldrich Chemicals (St. Louis, MO, USA), respectively. P5091 and imatinib were purchased Selleck Chemicals Inc. (Houston, TX, USA).

### Gene transfection

HEK293T cells at 50% confluence were subjected to gene transfection using polyethyleneimine (PEI) as the carrier as described previously^20^.

### Small interfering RNAs (siRNAs) and single-guided RNAs (sgRNA)

Specific siRNAs of USP7 from Ribobio (Guangzhou, Guangdong, China) were transfected into HEK293T cells using PEI as the gene carrier^19^. The two effective siRNAs were then chosen for further studies (target sequences, #1: 5′-GAATGACATGTACGAGAA-3′, #2: 5′-ACCCUUGGACAAUAUUCCU-3′). To knockdown USP7, two pieces of sgUSP7 were designed. The specific primers for sgUSP7 were: #1, 5′-CACCGAGTGATGGACACAACACCGC-3′ (F) and 5′-AAACGCGGTGTTGTGTCCATCACTC-3′ (R); #2, 5′-CACCGGGGATGGCAAATGGTGTAAA-3′ (F) and 5′-AAACTTTACACCATTTGCCATCCCC-3′ (R), provided by Sangon Biotech Inc., Shanghai, China.

### Western blotting (WB)

Collected cells were lysed on ice in a lysis buffer as described previously^21^, followed by centrifugation at high speed at 4 °C. The protein concentrations were then determined by the BCA assay (Beyotime). Equal amount proteins (30 µg) were fractionated in SDS-PAGE, and transferred to polyvinylidene difluoride membrane. The blots were subjected to analysis against appropriate antibodies as described previously^21^.

### Immunoprecipitation (IP)

Cell lysates from cells of interest were prepared in a RIPA buffer. After clarification and concentration determination, equal amount of total proteins were incubated with specific antibodies overnight at 4 °C followed by incubated with Protein A + G beads (Beyotime) for 1 h at room temperature. After wash, the protein-containing beads were collected and boiled in 2× SDS loading dye before being subjected to WB assays.

### CHX chase assay

After transfected with siRNA for 72 h, K562 cells were treated with CHX (100 μg/ml) for 0 to 12 h. Cell lysates were then prepared by 2× SDS lysis buffer, followed SDS-PAGE and WB analysis with specific antibodies as described previously^21^.

### Lentiviral USP7 construction

A human USP7 cDNA was inserted into the pLVX-AcGFP lentiviral vector (Clontech, Mountain View, CA, USA). The USP7 lentiviral particles were generated as described previously^19^.

### Constructs of BCR-ABL truncates

According to the domains, four truncate constructs of BCR-ABL (Fig. 3D) were cloned with specific primers as follows: for F1, 5′-CGGAATTCCTGTCGGGAATCCTGGCTAG-3′ (Forward) and 5′-GCTCTAGAATTGATGCTGGACAGGAAGT-3′ (Reverse); for F2, 5′-CGGAATTCGAGGAGATCACACCCCGACG-3′ (F) and 5′-GCTCTAGACTTGTCGTAGTTGGGGGACA-3′ (R); for F3, 5′-CGGAATTCTGGGAGATGGAACGCACGGA-3′ (F) and 5′-GCTCTAGAGCTGGAGCTGCCACCGCCCTC-3′ (R); for F4, 5′-CGGAATTCAAGCGCTTCCTGCGCTCTTG-3′ (F) and 5′-GCTCTAGACTACCTCTGCACTATGTCAC-3′ (R). The PCR was run as described previously^22^. After purification, the PCR products were cloned into a myc-tagged pcDNA3.1 vector between EcoRI and XbaI cloning sites.

### Site-directed mutagenesis of USP7

To generate an inactive USP7 plasmid, a C223S mutant was created by site-directed mutagenesis using wild-type USP7 as the template and the specific primers: 5′- GAGCGACTagtTACATGAACAGCCTGCTACAG-3′ (F) and 5′- CATGTAactAGTCGCTCCCTGATTCTTTAAGC-3′ (R). The site-directed mutagenesis was achieved by using the Mut Express II Fast Mutagenesis Kit V2 (Vazyme Biotech Co., Ltd, Nanjing, China) according to the instruction of the manufacturer^22^.

### Apoptosis detection with flow cytometric analysis

When cells were treated with ART or IM, cells were collected for Annexin V-FITC/PI staining as the instructions from the manufacturer (MultiSciences Biotech Co., Ltd, Hangzhou, China) and subjected to analysis on a BD flow cytometer as described previously^23^.

### Cell viability analysis by MTT assay

Leukemia cells were infected with lentiviral USP7 for 72 h before being subjected to MTT (3-(4,5-dimethylthylthiazol-2-yl)-2,5-diphenyltetrazolium bromide) assay as described previously^24^.

### Reverse-transcription polymerase chain reaction (RT-PCR)

Total RNA was extracted using Trizol^®^ (Transgene, Beijing, China). RNA (2.5 μg) was reverse-transcribed using an EasyScipt^®^ First-strand cDNA Synthesis SuperMix^®^ (Transgen Biotech Co., Ltd., Beijing, China) according to the manufacturer’s instruction. PCR amplification was performed using the following primers: for USP7, 5′-TTTTGTGCGAAATCTGCC-3′ (F) and 5′-AATCCCACGCAACTCCAT-3′ (R); for BCR-ABL, 5′-GAAGAAGTGTTTCAGAAGCTTCTCCC-3′ (F) and 5′-GACCCGGAGCTTTTCACCTTTAGTT-3′ (R); for GAPDH, 5′-AATCCCATCACCATCTTCC-3′ (F) and 5′-CATCACGCCACAGTTTCC-3′ (R). The PCR was run as described previously^22^. The PCR products were visualized by Goldview staining (Transgen) following electrophoresis on 2% agarose gels.

### Statistics

Statistical difference between the control and the experimental groups were analyzed by student’s *t*-test.

## Results

### USP7 stabilizes BCR-ABL

**Fig. 1:**
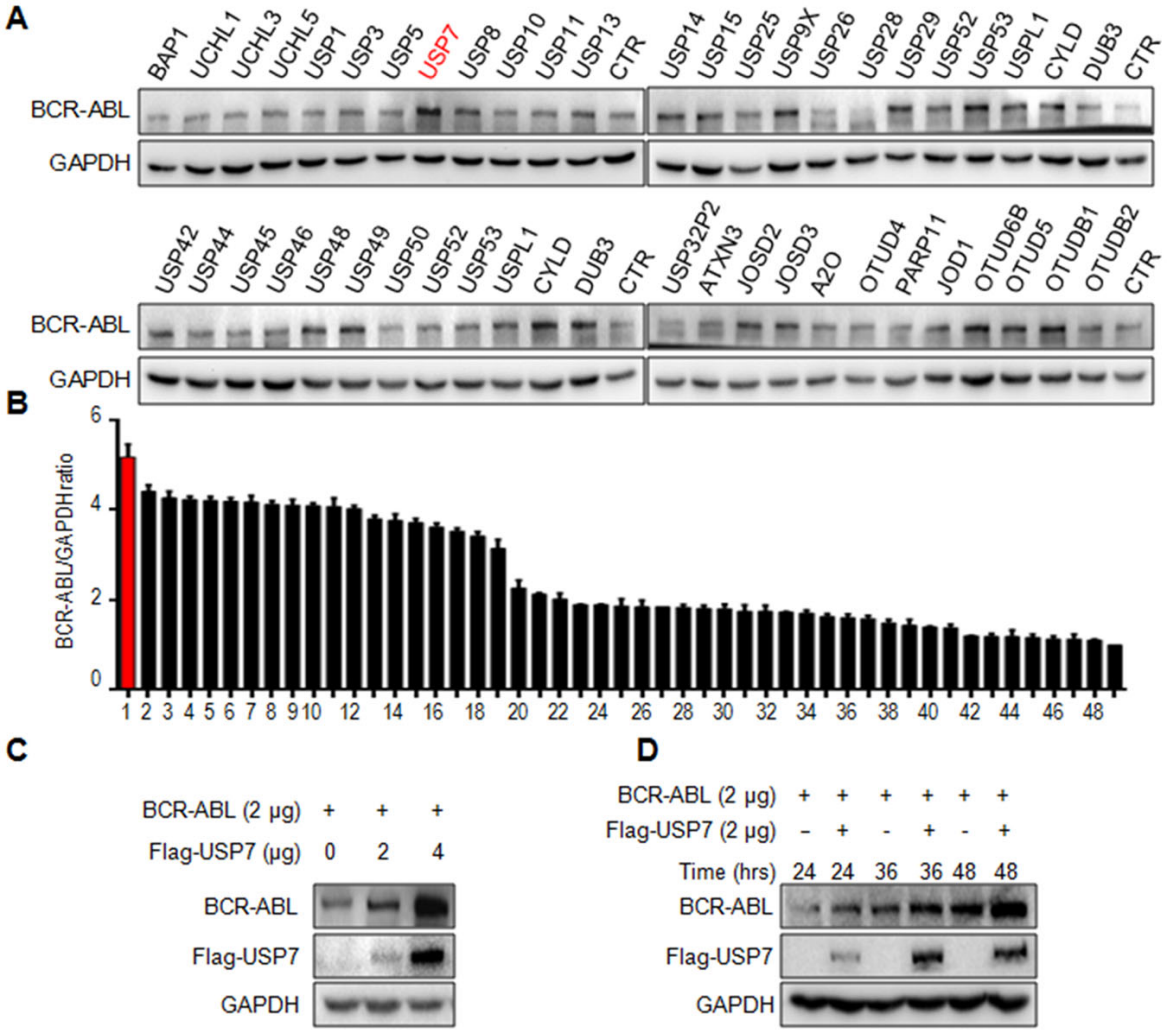
USP7 stabilizes BCR-ABL protein in CML cells. **A**, **B** HEK293T cells were transfected with individual deubiquitinases for 48 h, followed by WB assay to measure BCR-ABL at the protein levels. 1. USP7; 2. USP9X; 3. USP14; 4. USP8; 5. UCHL1; 6. USP1; 7. USP15; 8. USP13; 9. UCHL5; 10. USP5; 11. USP36; 12. USP25; 13. BAP1; 14. USP11; 15. USP10; 16. USP30; 17. UCHL3; 18. USP29; 19. USP38; 20. USP39; 21. USPL1; 22. USP49; 23. USP28; 24. CYLD; 25. OTUD4; 26. USP52; 27. USP46; 28. USP53; 29. DUB3; 30. USP44; 31. USP48; 32. USP42; 33. USP50; 34. ATXN3; 35. JOSD2; 36. USP45; 37. OTUD5; 38. USP26; 39. OTUB1; 40. USP3; 41. JOSD1; 42. OTUD6B; 43. USP32P2; 44. OTUB2; 45. JOD1; 46. JOSD3; 47. A2O; 48. PARP11; and 49. CTR. **C** HEK293T cells were transfected with BCR-ABL and USP7 plasmids as indicated for 48 h, followed cell lysate preparation and WB assays. **D** HEK293T cells were co-transfected BCR-ABL and USP7 or vector control for increasing periods before being harvested for WB.

**Fig. 2:**
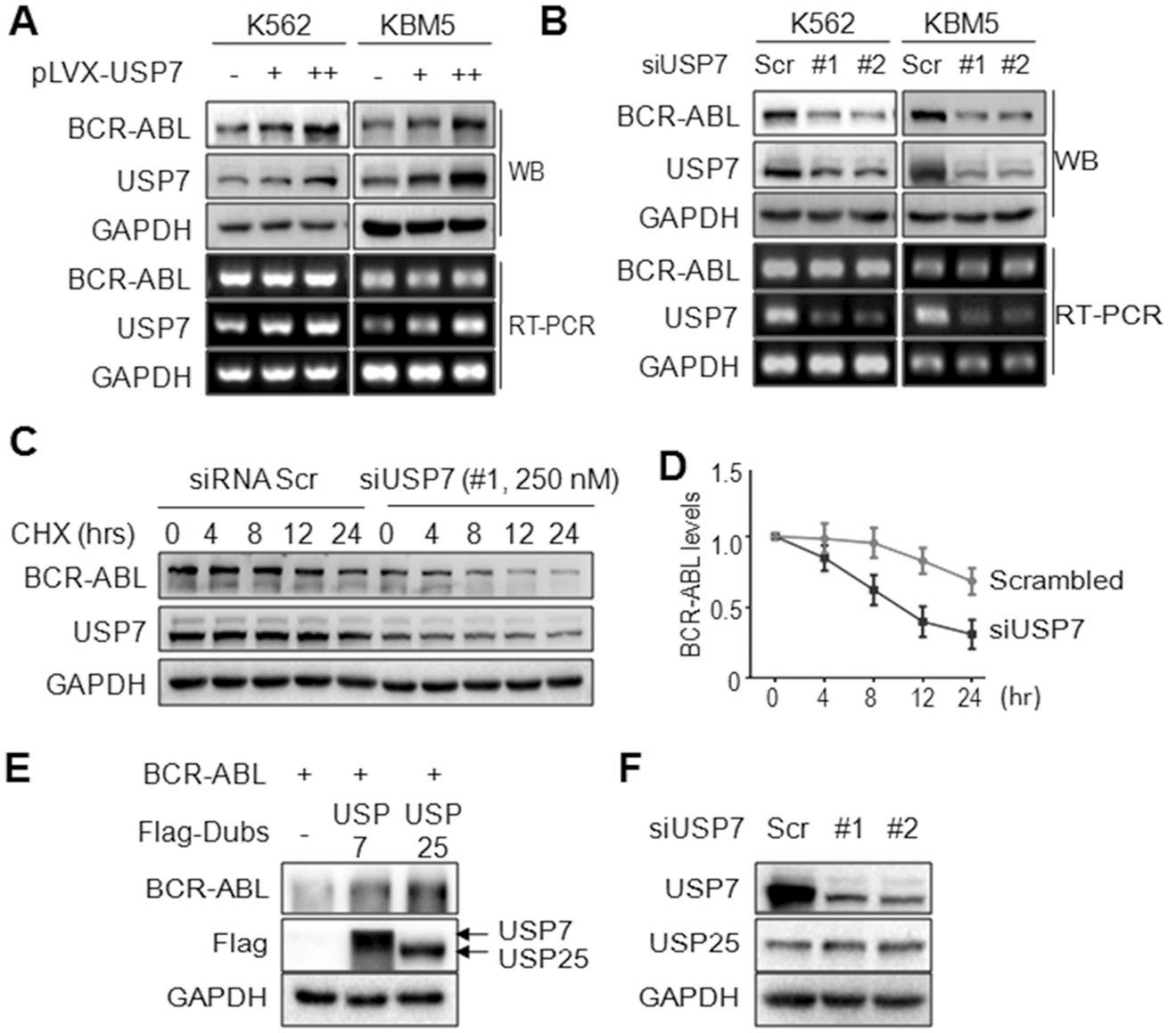
USP7 upregulates BCR-ABL in CML cells at the protein level. **A** K562 and KBM5 cells were infected with lentiviral USP7 for 96 h before being collected for WB and RT-PCR assays. **B** K562 and KBM5 cells were transfected with siUSP7 for 96 h, followed by WB and RT-PCR assays. **C** K562 cells were transfected with USP7 or scrambled siRNA for 96 h followed by CHX treatment for indicated periods, cell lysates were subjected to WB analysis. **D** The statistic analysis of CHX chase assay on BCR-ABL/GAPDH in **C**. **E** The BCR-ABL plasmid was co-transfected with USP7 or USP25 plasmids into HEK293T cells, 24 h later, cells were collected for WB against indicated proteins. **F** siUSP7 was transfected into K562 cells for 96 h, followed by WB against indicated proteins. Scr scramble.

BCR-ABL is a determinant factor in the pathophysiological process of CML, and downregulation of BCR-ABL leads to CML cell death. BCR-ABL degradation is processed via the ubiquitin-proteasomal pathway under the direction of ubiquitin ligases including CHIP and SH2-U-box^11,12^. USP25 has been reported to stabilize BCR-ABL in a recent study^13^. To identify more potential Dubs for BCR-ABL, we screened a 48-Dub library against BCR-ABL by WB assays and found that USP7 was the most potent one to increase the BCR-ABL protein level (Fig. 1A, B). To confirm this finding, BCR-ABL and USP7 plasmids were co-transfected into HEK293T cells by increasing the plasmid concentrations or the incubation time. The subsequent assays indicated that the BCR-ABL protein was stabilized by USP7 in a concentration- and time-dependent manner (Fig. 1C, D). This finding was confirmed in human CML cell lines K562 and KBM5. As shown in Fig. 2A, overexpression of USP7 led to a marked increase of the BCR-ABL protein. In contrast, when USP7 was knocked down, the BCR-ABL protein was downregulated accordingly (Fig. 2B). Notably, USP7 markedly altered BCR-ABL at the protein level but not at the mRNA level (Fig. 2A, B). These results were further confirmed by CHX chase assay as shown in Fig. 2C, D. When the de novo synthesis of the BCR-ABL protein was inhibited by CHX, knockdown of USP7 led to a shorter half-life of BCR-ABL than the mock control. Because USP25 has been reported as a Dub of BCR-ABL, we therefore compared the activity of USP7 and USP25 towards BCR-ABL protein stability. The result showed that both enzymes stabilized BCR-ABL (Fig. 2E). To find out whether USP7 activity was interfered by USP25, we knocked down USP7 from K562 cells, followed by measuring the expression level of both USP7 and USP25, it turned out the USP7 but not USP25 was downregulated by siUSP7 (Fig. 2F). Thus, these results collectively demonstrated that USP7 stabilizes BCR-ABL.

### USP7 interacts with and deubiquitinates BCR-ABL

**Fig. 3:**
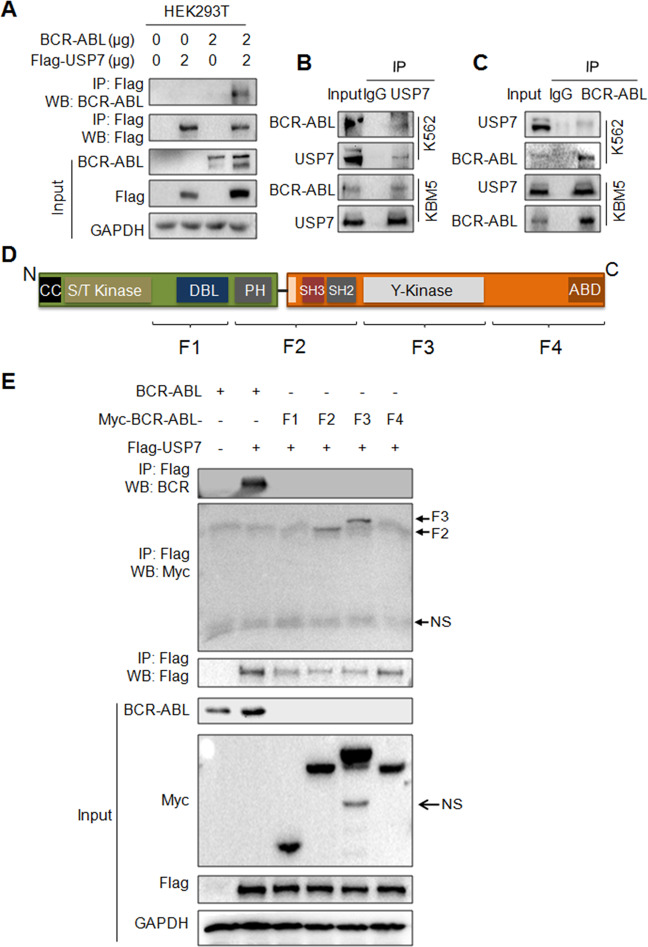
USP7 interacts with BCR-ABL via both SH3-SH2 and Y-kinase domains. **A** HEK293T cells were transfected with BCR-ABL and/or USP7 plasmids for 48 h. Cell lysates were then prepared for IP/WB assays as indicated. **B**, **C** Cell lysates from K562 and KBM5 were immunoprecipitated with an anti-USP7 antibody (**B**) or an anti-BCR-ABL antibody (**C**) before being applied for WB assays. **D** The schematic draw of BCR-ABL structure. CC coiled-coil domain, ABD actin-binding domain. **E** HEK293T cells were co-transfected with a USP7 plasmid and BCR-ABL constructs as indicated for 24 h, the cell lysates were subjected to IP/WB assays as indicated. NS not specific.

Given that USP7 is a Dub and it stabilizes BCR-ABL, we next wondered whether USP7 could deubiquitinate BCR-ABL. To this end, we first examined the interaction between these two proteins in both the tool cell line HEK293T and CML cell lines K562 and KBM5. The results showed that BCR-ABL could be identified in the USP7 immunoprecipitates (Fig. 3A, B). To confirm this finding, we also performed a immunoprecipitation using an anti-BCR-ABL antibody followed by WB assays using USP7 as the primary antibody. The result showed that USP7 was also detected in the BCR-ABL interacting proteins from CML cells (Fig. 3C). Moreover, we analyzed the structure of BCR-ABL and made several constructs composed different domains as shown in Fig. 3D. These domain-specific constructs and full-length BCR-ABL were co-transfected with USP7 into HEK293T cells, followed by IP/WB assay. The results showed that full-length (wild-type) BCR-ABL and two truncates including that contains the linking region of BCR-ABL and that contains the Y kinase domain were found in the USP7 immunoprecipitates, suggesting the linking region and the Y kinase domain of BCR-ABL might be critical for its interaction with USP7 (Fig. 3E). Therefore, USP7 and BCR-ABL interacted each other in both ectopic and endogenous contexts, which was consistent with a previous report^25^.

**Fig. 4:**
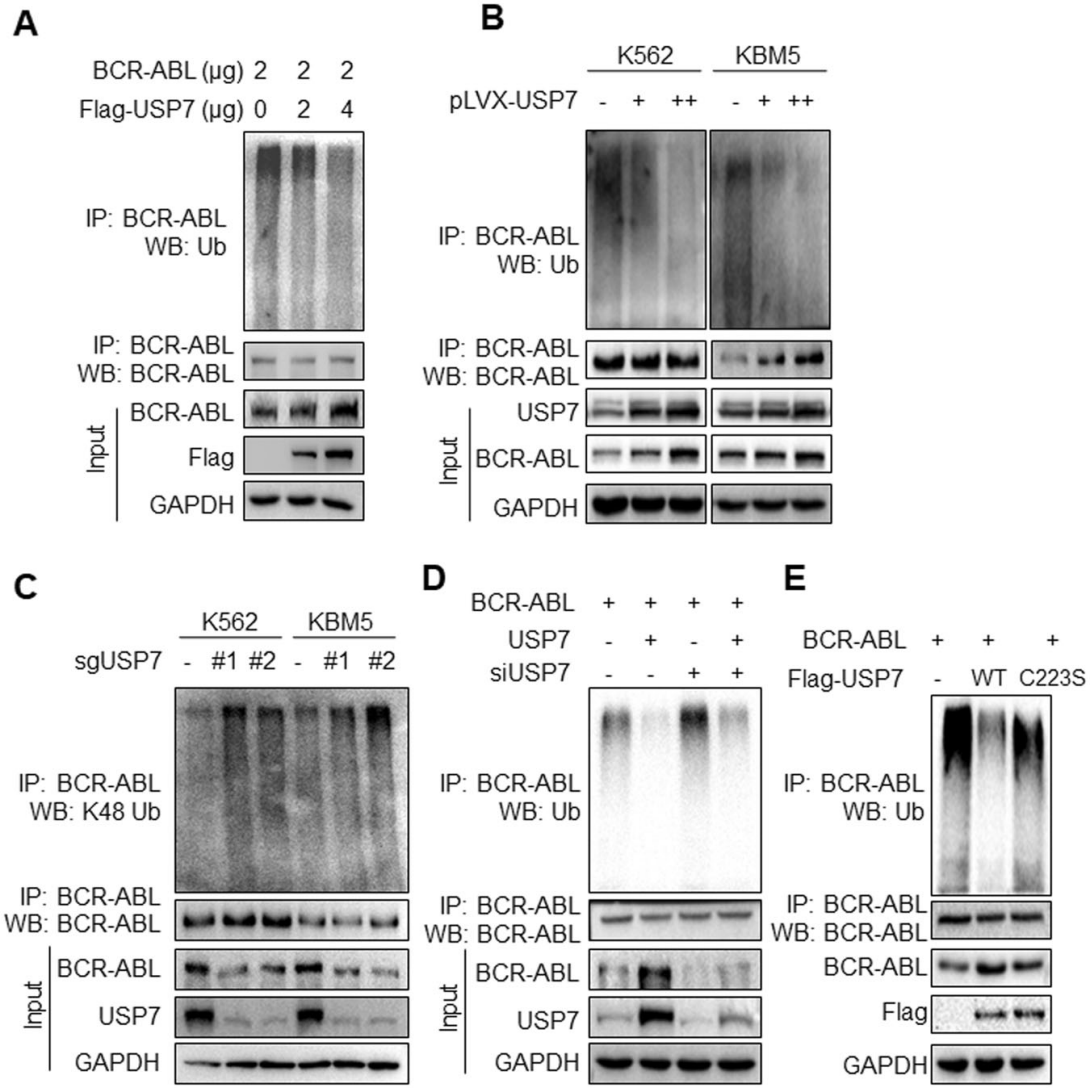
USP7 deubiquitinates BCR-ABL in CML cells. **A** HEK293T cells were transfected with BCR-ABL and/or USP7 plasmids for 48 h. Cell lysates were then prepared for IP/WB assays as indicated. **B** K562 and KBM5 were infected with lentiviral USP7 for 96 h, followed by cell lysate preparation and IP/WB assays as indicated. **C** CML cell lines were infected with sgUSP7 for 96 h, followed by cell lysate preparation and IP/WB assays as indicated. **D** USP7 was knocked out by the specific siRNA and or USP7 plasmids. Forty-eight hours later, cell lysates were then prepared for IP/WB assays as indicated. **E** HEK293T cells were transfected with BCR-ABL and WT or C223S USP7 plasmids. Twenty-four hours later, cell lysates were prepared for an IP/WB assay as indicated.

Since USP7 functions as a Dub, we wondered it might regulate BCR-ABL stability through deubiquitination. To verify this hypothesis, the USP7 plasmid was co-transfected into HEK293T cells with BCR-ABL, followed by IP/WB assays. The results showed that USP7 significantly decreased the polyubiquitination level of BCR-ABL in association with its stabilized protein (Fig. 4A). This finding was further confirmed in CML cells. As shown in Fig. 4B, lentiviral USP7 markedly decreased the ubiquitination levels of BCR-ABL in both K562 and KBM5 cell lines. Moreover, we found that USP7 could prevent BCR-ABL from K48-linked polyubiquitination because when USP7 was knocked out, the K48-linked polyubiquitination levels of BCR-ABL were markedly increased (Fig. 4C). We further evaluated the ubiquitination level of BCR-ABL when USP7 was rescued after knocked down. And we found that USP7 prevented BCR-ABL from polyubiquitination and it was largely decreased when USP7 was knocked down by its specific siRNA (Fig. 4D). Moreover, it is reported that C223 is essential for the deubiquitinating activity of USP7^26^, to further evaluate the effects of USP7 on BCR-ABL ubiquitination, we made a C223S mutant USP7 construct and found that it inactivated USP7. Compared with the wild-type one, the C223S mutant failed to deubiquitinate BCR-ABL (Fig. 4E). Taken together, these data concluded that USP7 interacted with BCR-ABL and stabilized its protein levels by decreasing its K48-linked polyubiquitination levels, a major ubiquitination manner in modulating protein stability.

### USP7 activates the BCR-ABL signaling pathway

**Fig. 5:**
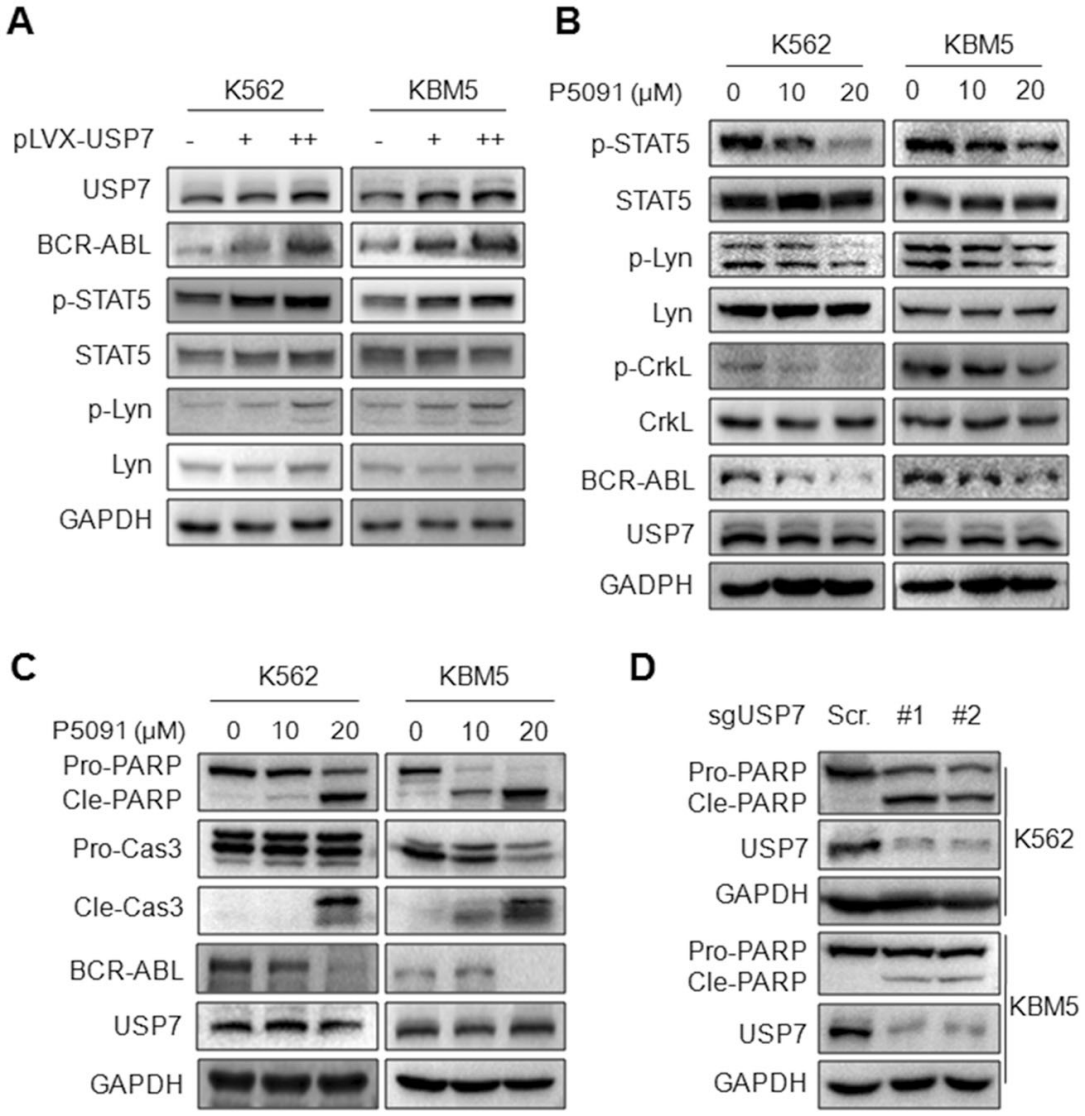
USP7 activates the BCR-ABL signaling pathway in CML cells. **A** K562 and KBM5 cells were infected with USP7 lentivirus for 96 h before being collected for WB assays against specific proteins as indicated. **B**, **C** K562 and KBM5 cells were treated with P5091 for 24 h, followed by WB assays as indicated. **D** K562 and KBM5 cells were infected with sgUSP7 lentivirus for 96 h before being collected for WB assays against indicated proteins.

BCR-ABL is a non-receptor tyrosine kinase that activates a series of signal cascades to promote CML oncogenesis and progression^4^. Among these a key role is played by STAT1 and STAT5 that are constantly active in BCR-ABL–positive cell lines and in primary cells from CML patients^4^. We therefore examined the effects of USP7 on STAT5 phosphorylation. As shown in Fig. 5A, ectopic expression of USP7 led to an increased activation of STAT5. Moreover, USP7 also increased the phosphorylation of Lyn, another non-receptor tyrosine kinase and a component of the BCR-ABL signaling complex^27^ (Fig. 5A). We also evaluated this effect by P5091, a specific USP7 inhibitor^19,28^. As shown in Fig. 5B, P5091 significantly decreased STAT5, Lyn and CRKL phosphorylation. Notably, all these effects were closely associated with BCR-ABL stability (Fig. 5A). Given the activation of the BCR-ABL signaling pathway is critical for BCR-ABL to promote CML cell survival, we next measured CML cell apoptotic signals after P5091 treatment. The result showed that inhibition of USP7 by its inhibitor P5091 activated both PARP and Caspase-3 cleavage (Fig. 5C), the hallmark of apoptosis. We next wondered whether knockdown of USP7 could result in CML cell apoptosis, to this end, CML cells were infected with sgUSP7 lentivirus for 96 h, followed by WB assay. It showed that sgUSP7 induced the cleavage of PARP (Fig. 5D), suggesting knockdown USP7 leads to CML cell apoptosis. Therefore, USP7 stabilizes BCR-ABL and activates its signaling pathway and inhibition of USP7 activates the apoptotic signals in CML cells.

### USP7 promotes CML cell survival

**Fig. 6:**
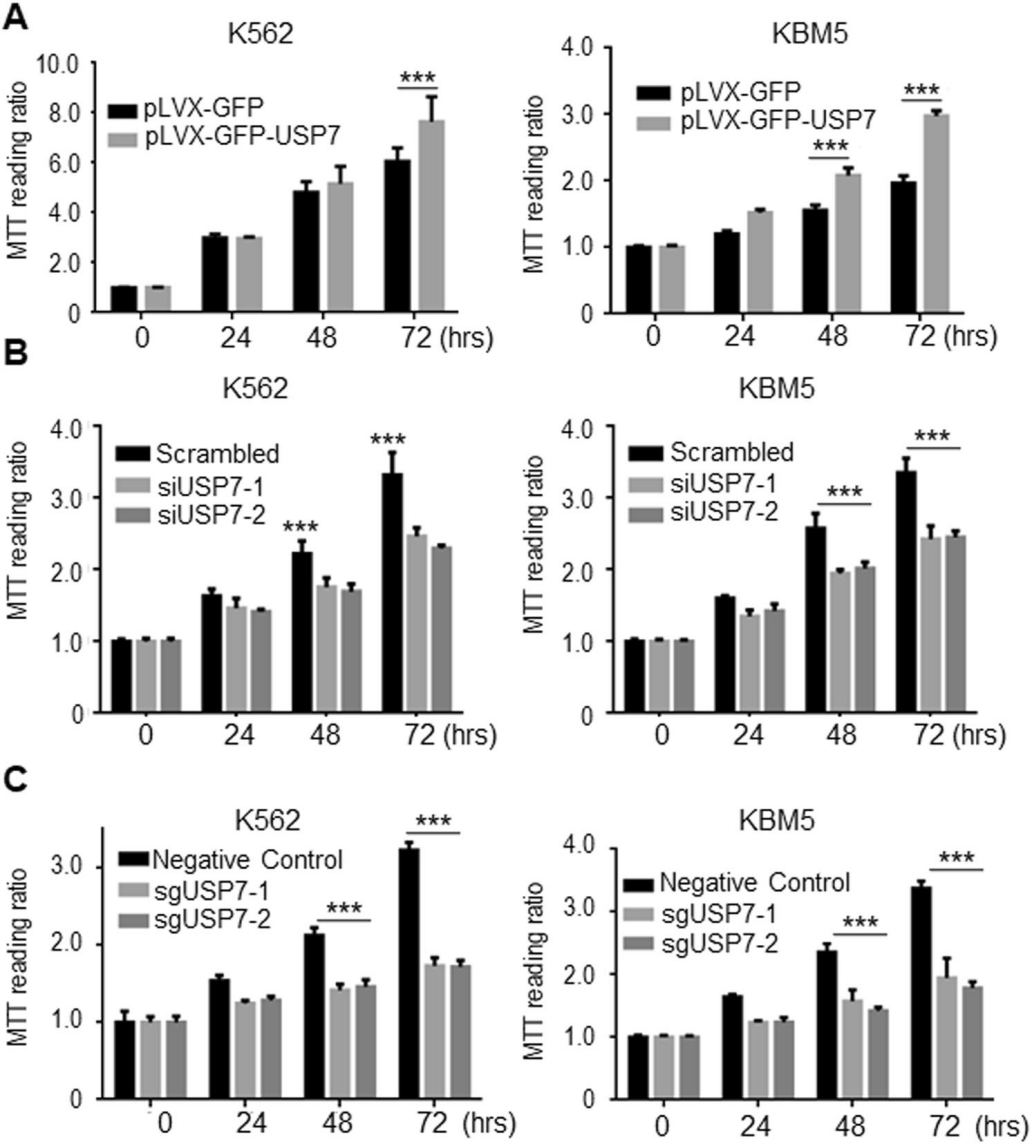
USP7 increases CML cell survival. **A** K562 and KBM5 cells were infected with lentiviral USP7 for 72 h, followed by MTT assay for the indicated periods. **B** K562 and KBM5 cells were knocked down USP7 by specific siRNAs for 72 h, followed by MTT assay for the indicated periods. **C** K562 and KBM5 cells were infected with lentiviral sgUSP7 for 72 h, followed by MTT assay for the indicated periods.

The aforementioned studies demonstrated that USP7 acted as a Dub for BCR-ABL ubiquitination and a regulator of BCR-ABL-related oncogenic signaling pathways, suggesting USP7 might promote CML cell survival. To investigate this effect, CML cell lines were infected by lentiviral USP7 followed by cell viability evaluation using MTT assay. As shown in Fig. 6A, lentiviral USP7 markedly increased MTT readings of both K562 and KBM5 cells. In line with it, knockdown of USP7 decreased those MTT readings (Fig. 6B). Moreover, we knocked out USP7 by its specific sgRNA, the resultant assay showed that MTT readings were also significantly decreased (Fig. 6C), very similar to those from siUSP7 transfection (Fig. 6B) and consistent to the hallmark cleavage of PARP that reflected cell apoptosis (Fig. 5D). Given MTT assay is a comprehensive evaluation of viable cells resulted from proliferation and survival, these studies showed that USP7 promotes CML cell survival and/or proliferation.

### ART downregulates BCR-ABL signaling and induces CML cell apoptosis

**Fig. 7:**
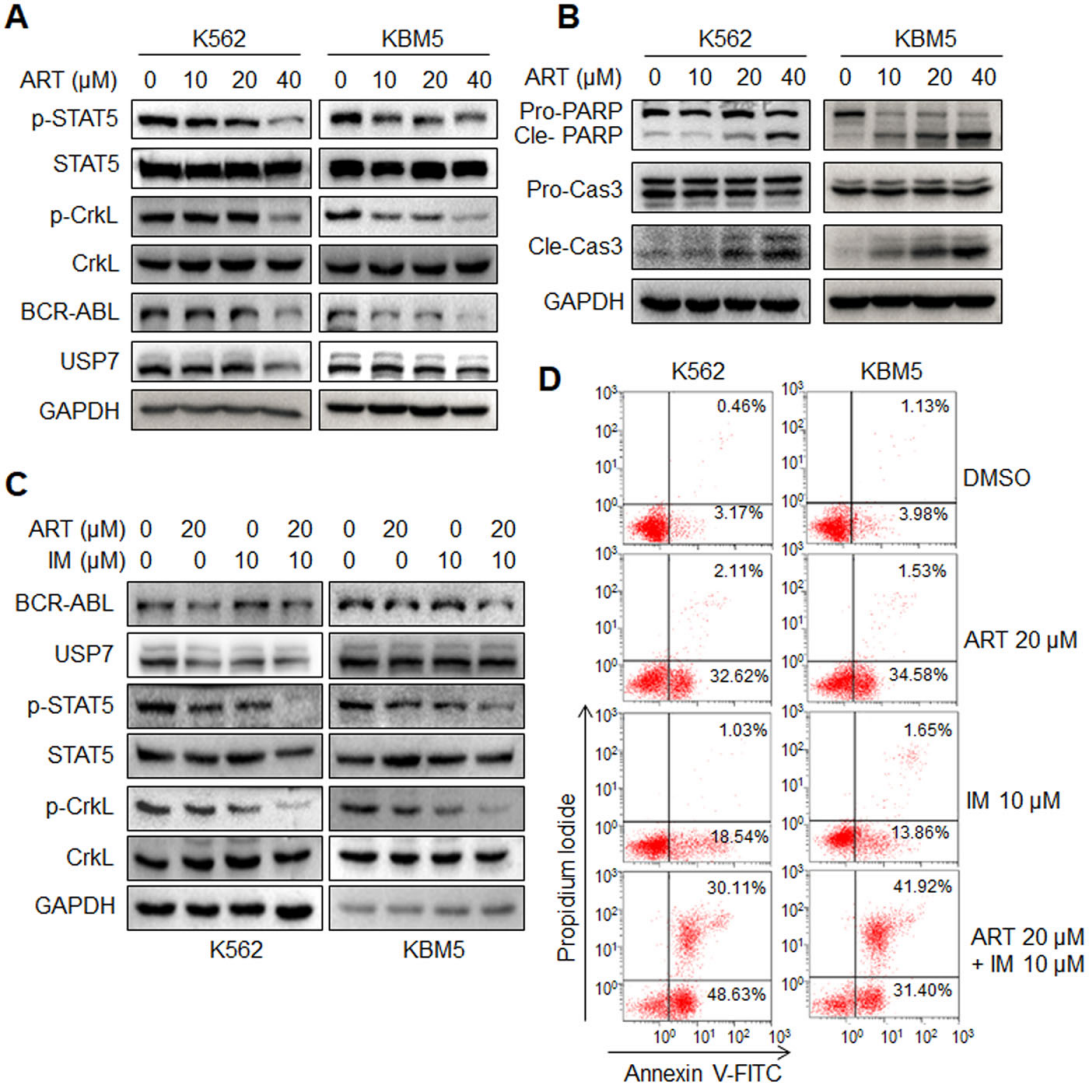
Artesunate suppresses BCR-ABL signaling transduction and enhances CML cell apoptosis with imatinib. **A**, **B** K562 and KBM5 cells were treated with ART for 24 h, followed by WB analysis with the specific antibodies. **C** K562 and KBM5 cells were treated with ART and/or imatinib (IM) for 24 h, and followed by WB analysis with the specific antibodies. **D** K562 and KBM5 cells were treated with ART and/or imatinib (IM) for 24 h, and followed by Annexin V-FITC/PI staining and flow cytometric analyses.

The drug resistance of tyrosine kinase inhibitors (TKIs) becomes a critical challenge for the treatment of CML^29^, it is thus urgent to find novel anti-CML drugs by targeting BCR-ABL. In the screen for anti-CML drugs, ART, the antimalarial drug, was turned out as one of the most potent candidates. ART downregulated BCR-ABL protein, along with the phosphorylation of both STAT5 and CRKL (Fig. 7A). Moreover, ART induced significant apoptosis of CML cell lines evidenced as PARP and Caspase-3 cleavage (Fig. 7B), consistent with a previous report^30^. Furthermore, ART displayed potent synergistic effects with imatinib (IM), a specific inhibitor of BCR-ABL, in terms of the BCR-ABL signaling transduction. As shown in Fig. 7C, ART strikingly enhanced the inhibitory effects on STAT5 and CRKL activation as assayed by their phosphorylation levels. Moreover, ART markedly increased CML cell apoptosis induced by IM. For example, IM at 10 µM and ART at 20 µM induced only 19% and 34% of cell death, respectively, in K562 cells, but the combined treatment resulted in more than 78% cell death. And the similar effect was also observed in KBM5 cells (Fig. 7D). These data suggested ART might act on USP7/BCR-ABL therefore inducing CML cell apoptosis.

### ART induces BCR-ABL degradation by accumulating its ubiquitination

**Fig. 8:**
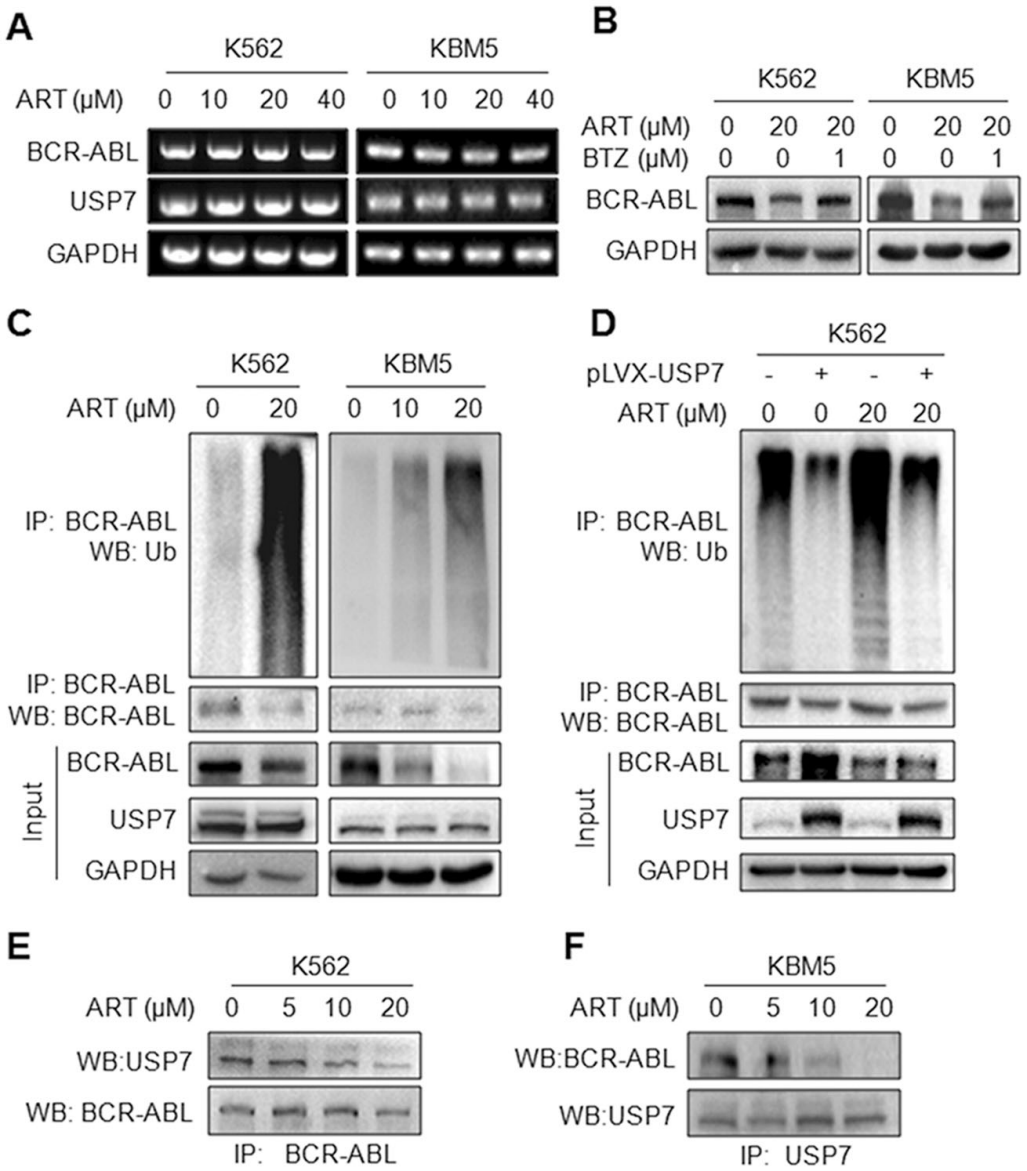
Artesunate induces BCR-ABL degradation by disrupting its interaction with USP7. **A** After being treated with ART for 24 h, K562 and KBM5 cells were subjected to WB and RT-PCR assays as indicated. **B** K562 and KBM5 cells were treated with ART for 12 h, followed by bortezomib (BTZ) or vehicle control treatment for another 12 h before being prepared for WB analysis. **C** K562 and KBM5 cells were treated with ART for 24 h, and the cell lysates were subjected to IP/WB assays with indicated antibodies to measure BCR-ABL ubiquitination levels. **D** K562 cells were infected with lentiviral USP7 for 72 h, followed by ART treatment for another 24 h before being prepared for IP/WB assays. **E**, **F** K562 and KBM5 cells were treated with ART for 24 h, followed by IP/WB assays with indicated antibodies.

Given BCR-ABL stability is modulated by USP7 and ART downregulates BCR-ABL protein, we next wondered whether ART acted on the USP7/BCR-ABL axis. Firstly, we evaluated the effects of ART on BCR-ABL expression at the transcriptional level. In contrast to the changes at the protein levels (Fig. 7A), ART showed no marked effects on the mRNA levels of BCR-ABL (Fig. 8A), indicating ART might modulate BCR-ABL protein through posttranscriptional modifications. Therefore, we next examined the BCR-ABL protein stability by ART in the presence of bortezomib (BTZ), a typical inhibitor of the proteasomes. It turned out that ART-induced BCR-ABL degradation was blocked by BTZ (Fig. 8B), implicating that ART might promote BCR-ABL degradation via the UPP. To elucidate this hypothesis, K562 and KBM5 cells were treated with ART, followed by IP/WB assay. As shown in Fig. 8C, the results showed that ART increased BCR-ABL ubiquitination levels (upper panel) and promoted its degradation (lower panel). To confirm the exact role of ART on USP7/BCR-ABL axis, K562 cells were treated with ART alone or together with infection of lentiviral USP7, followed by IP/WB assays for BCR-ABL ubiquitination. As showed in Fig. 8D, ectopic expression of USP7 significantly reduced BCR-ABL polyubiquitination but it was markedly recovered by ART, suggesting ART acts against USP7. Because the previous study showed that USP7 binds to and deubiquitinates BCR-ABL, we wondered to know whether ART disrupts this interaction. To this end, K562 cells were treated with increased ART, followed by IP/WB assays in which IP with anti-BCR-ABL and WB with anti-USP7. As shown in Fig. 8E, ART decreased the presence of USP7 in the BCR-ABL complex in a concentration-dependent manner. Furthermore, we performed another assay in KBM5 cells in which the IP and WB assays were performed with anti-USP7 and anti-BCR-ABL antibodies, respectively. The result showed that the presence of BCR-ABL was markedly reduced from the USP7 precipitates by ART in a concentration-dependent manner (Fig. 8F). Therefore, ART might disrupt the USP7/BCR-ABL interaction and accumulates BCR-ABL ubiquitination that leads to BCR-ABL degradation in the UPP.

## Discussion

BCR-ABL is a notorious protein in CML pathophysiology that initiates and promotes CML by activating multiple signals as an oncogenic non-receptor tyrosine kinase. Multiple mutations in BCR-ABL is also a notorious obstacle in the targeted therapy of CML. Therefore, suppression of BCR-ABL is urgent for eradicating the disease. It is clearly known that the BCR-ABL protein is processed by proteasomes after K48-linked polyubiquitination under the direction of ubiquitin ligases such as CHIP^11^, c-CBL, and SH2-U-box^12^. The present study found that the Dub USP7 stabilizes BCR-ABL and the USP7/BCR-ABL could be an ideal target for CML treatment.

Known as ubiquitin carboxyl-terminal hydrolase 7 or herpes virus-associated ubiquitin-specific protease (HAUSP), USP7 is a cysteine protease that cleaves the isopeptide bond between the C terminus of ubiquitin and the ε-amine of the target lysine. Several tumor-associated proteins have been identified as USP7 substrates, including MDM2/p53^28^, FOXO4^31^, PTEN^32^, and c-Maf^19^. The present study added BCR-ABL as a novel substrate of USP7. As shown in our study, USP7 interacts with BCR-ABL and prevents it from K48-linked polyubiquitination therefore stabilizing its protein but not altering its mRNA. Interestingly, previous reports have shown the association between BCR-ABL and USP7, but these studies do not identified USP7 as a Dub of BCR-ABL, in contrast, they found USP7 is a kinase substrate of BCR-ABL because BCR-ABL phosphorylates USP7 and triggers its Dub activity toward PTEN resulting in PTEN nuclear exclusion after deubiquitination^25^. The present study found that USP7 interacts with the Y-kinase domain of BCR-ABL (Fig. 3E) which probably explains BCR-ABL phosphorylates USP7^25^. Moreover, USP7 can stabilize BCR-ABL, therefore, USP7 and BCR-ABL might form a positive feedback in that USP7 stabilizes BCR-ABL, and BCR-ABL phosphorylates and activates USP7, therefore further promoting CML pathophysiology.

In addition to USP7, another Dub, USP25, was recently reported to deubiquitinate and stabilize BCR-ABL^13^. USP25 was also found as one of the top Dub candidates that stabilize BCR-ABL (Fig. 1A, B). Our study showed there are no crosstalk between USP7 and USP25 because USP7 knockdown leads no changes in USP25 expression (Fig. 2F). Moreover, USP9x might also act as a Dub of BCR-ABL. But different from USP7 and USP25 that remove K48-linked polyubiquitination from BCR-ABL, USP9x strips K63-linked polyubiquitination of BCR-ABL. Inhibition of USP9x by a small molecule chemical WP1130 results in a rapid accumulation of K63-linked ubiquitin polymer on BCR-ABL. WP1130 does not bring BCR-ABL to degradation, in contrast, it induces BCR-ABL aggresomes and inhibits its oncogenic kinase activity^15^. Our previous study found USP10 is also associated with BCR-ABL but in an indirectly manner in which USP10 acts as a Dub of the ubiquitin ligase SKP2 that mediates K63-linked ubiquitination of BCR-ABL thus enhancing its kinase activity^33^. Therefore, this present study identifies USP7 as a Dub of BCR-ABL that stabilizes BCR-ABL by preventing it from K48-linked polyubiquitination that leads to protein degradation in proteasomes.

The pharmacological significance in the present study is that we found that overexpression of USP7 promotes CML cell survival while knockdown of USP7 decreases CML cell viability. This result leads to the discovery of ART that can disrupt the interaction between USP7 and BCR-ABL therefore accumulating BCR-ABL polyubiquitination and promoting its degradation. Notably, ART shows potent synergistic effects with imatinib, the specific inhibitor of BCR-ABL kinase and the major anti-CML drug, to induce CML cell apoptosis.

Collectively, our study identifies USP7 is a novel Dub of BCR-ABL and a promoter of BCR-ABL signaling pathway. Targeting USP7/BCR-ABL might be a potential strategy for CML treatment.
